# Oxygen-enriched oleic matrix (NovoX) for wound healing in pediatric patients undergoing open surgical treatment for pilonidal disease: Preliminary experience

**DOI:** 10.3389/fped.2022.1068280

**Published:** 2022-11-25

**Authors:** Marta Bisol, Sophia Tykhomyrova, Camilla Pagliara, Maria-Grazia Scarpa, Edoardo Guida, Damiana Olenik, Daniela Codrich, Jürgen Schleef, Alessandro Boscarelli

**Affiliations:** ^1^ Division of Pediatric Surgery, Department of Women’s and Children’s Health, University of Padua, Padua, Italy; ^2^ Municipal Non-Profit Enterprise “Lviv Territorial Medical Union”, Multidisciplinary Clinical Hospital of Emergency and Intensive Care - Separated Division “Hospital of Saint Nicolaus”, Danylo Halytsky Lviv National Medical University, Lviv, Ukraine; ^3^ Department of Pediatric Surgery and Urology, Institute of Maternal and Child Health - IRCCS “Burlo Garofolo”, Trieste, Italy; ^4^ Chief of Surgical Department, Institute for Maternal and Child Health - IRCCS “Burlo Garofolo”, Trieste, Italy

**Keywords:** sinus pilonidalis, children, post operative treatment, oxygen enrichment therapy, wound healing

## Abstract

**Introduction:**

Pilonidal disease (PD) is a common infectious and inflammatory condition affecting the gluteal cleft and sacrococcygeal region. The optimal treatment for PD remains controversial. While the open technique reduces the number of relapses compared to minimally invasive approaches, it is associated with a longer healing time. Reactive oxygen species are a key part of the normal wound-healing process. Herein, we reported our preliminary experience using a new oxygen-enriched oil-based product called NovoX for wound healing after open surgery for PD.

**Materials and methods:**

We used a new oxygen-enriched product for wound healing in three pediatric patients undergoing open surgical repair for PD between December 2021 and April 2022. During postoperative follow-up, healing time and the aesthetic result were evaluated.

**Results:**

Our preliminary study included three patients with chronic PD. The average follow-up time was 5 weeks, corresponding to the end of the healing process and the resumption of normal daily activities. Only one mild complication occurred during the study period. No short-term side effects were reported. The cosmetic result was reported as satisfactory.

**Conclusion:**

NovoX is easy to apply, safe, and effective for treating pediatric patients undergoing open surgical treatment for PD, leading to slightly faster wound healing with good aesthetic outcomes.

## Introduction

Pilonidal disease (PD) is a condition that typically affects the gluteal cleft and sacrococcygeal region in adolescents and young adults. While the pilonidal condition spectrum in children includes pilonidal cysts, abscesses, pits, and sinuses, PD is commonly characterized by sacrococcygeal abscesses that form due to a chronic inflammatory response to hair follicle retention in the intergluteal subcutaneous tissue. PD occurs more frequently in male patients. Pain, bleeding, and recurrent secretions are the most representative symptoms of acute disease, often requiring several interventions and negatively impacting patients’ quality of life. PD treatment remains controversial, with many surgical techniques having different success rates and no agreed-upon gold standard approach (1–3).

Innovative medical devices have been developed for use in postoperative wound healing management. Numerous lines of evidence show that wound healing is impaired in hypoxic conditions and that reactive oxygen species (ROS) are a key component of the normal wound-healing response. Recently, a new oxygen-enriched oil-based product called NovoX (Moss SpA; Lesa, Novara, Italy) was shown to create a local microenvironment unfavorable to pathogen growth and to promote the healing of deep and narrow wounds, including those secondary to surgical procedures. NovoX is an approved class IIb Medical Device in the European Union, which is on the market since 2011 and is currently CE marked according to the EU Regulation 2017/745 (MDR) (4–6).

Herein, we describe our preliminary experience using NovoX for wound healing in pediatric patients undergoing open surgery for PD.

## Materials and methods

Patients referred to our Pediatric Surgical Center between December 2021 and April 2022 for PD were evaluated for inclusion in this study. Surgical procedures were performed by three different experienced surgeons as follows. Under general anesthesia and prone position, routine skin preparation was performed, and sterile drapes were placed after a few minutes according to our safe surgery guidelines. Methylene blue was initially injected in the fistulous orifices to define disease spread. Therefore, the pathologic tissue is removed *en bloc* with a diamond-shaped incision downward to the fascia underneath. The wound is partially closed with interrupted resorbable sutures or kept completely open and medicated. All patients enrolled were medicated with NovoX directly applied to the wound and covered with a hyaluronic acid gauze. Follow-up evaluations were scheduled with the surgeon who performed the procedure. Our follow-up protocol included a weekly outpatient evaluation for the first 2 months. During each visit, the surgeon accurately examined the wound, evaluating the healing process and documenting possible complications and cosmetic results with photographs.

## Results

Three patients with chronic PD were enrolled in this preliminary study. They comprised two males and one female with a median age at surgery of 16.3 years. Two patients suffered from severe obesity with an average weight and body mass index of 107.3 and 35.3, respectively. One patient presented with a history of suppurative hidradenitis and Hashimoto's thyroiditis. Another patient suffered from IperIgD disease, insipid diabetes, and mevalonate kinase deficiency. In all cases, a recurrent pilonidal sinus disease was described. One patient required a pilonidal abscess incision 3 months before surgical treatment, while the other two patients were treated with antibiotic therapy before surgery.

**Figure 1:**
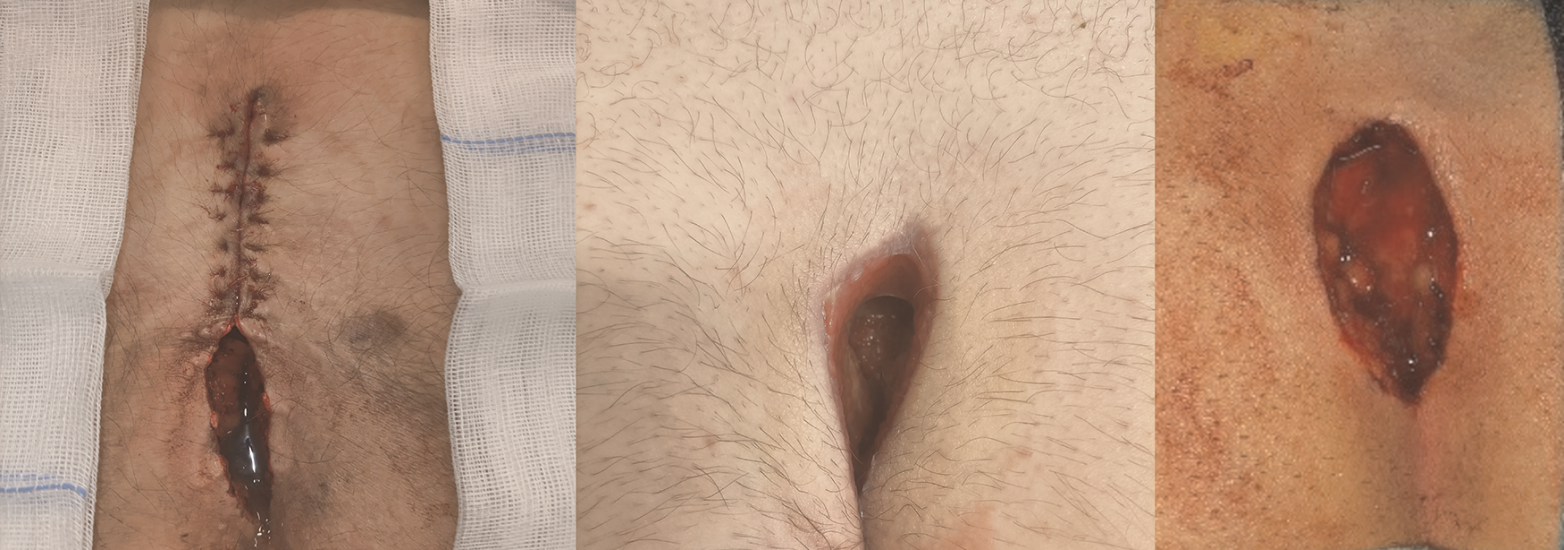
Close-up views of our three patients’ surgical wounds immediately after open midline treatment of chronic PD. NovoX was applied directly inside the wounds.

All patients underwent definitive surgical excision of a pilonidal cyst using the open surgical technique. Surgery length ranged from 30 to 85 min. The wound was partially closed in one patient with interrupted stitches in resorbable material. The wound was kept totally open in the other two cases. No intraoperative complications were described. Two cases were medicated with NovoX gel and hyaluronic acid gauze. In the last patient, NovoX roll and hyaluronic acid gauze were used immediately after the operation and then replaced with NovoX gel (Figure 1). Their median hospitalization period was 1.6 days.

**Figure 2:**
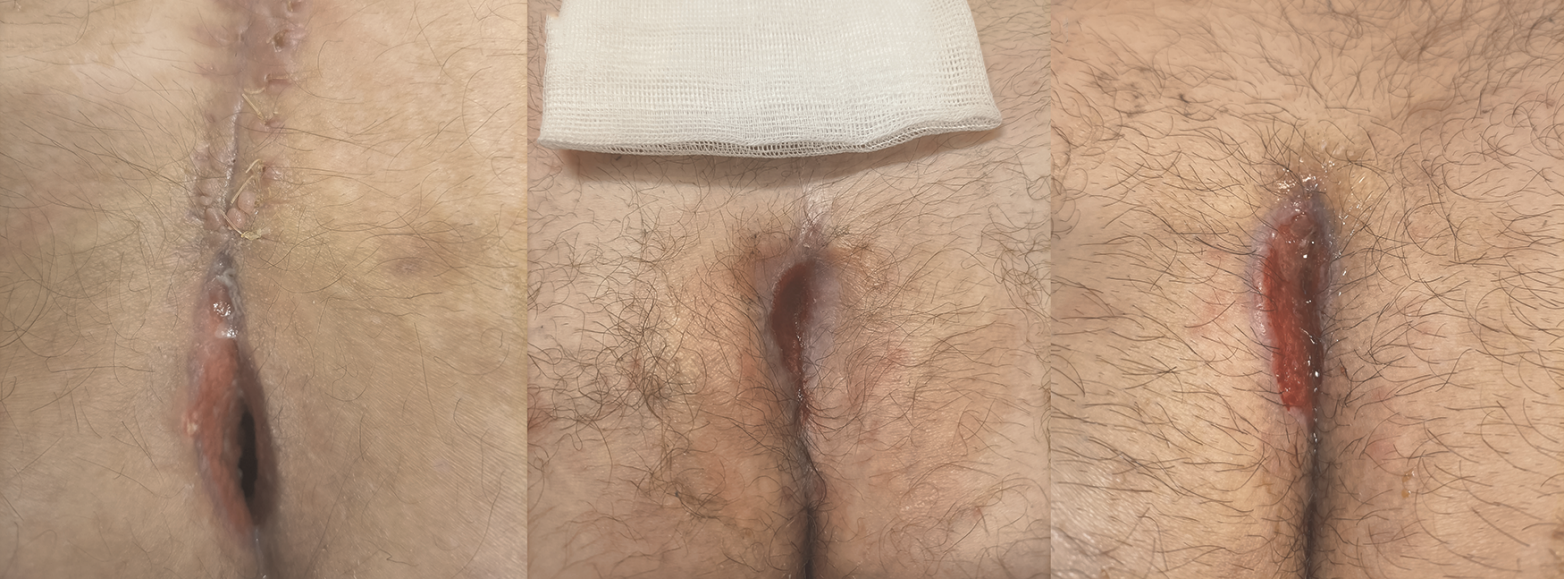
Surgical wounds’ clinical appearance after 3 weeks of NovoX treatment.

**Figure 3:**
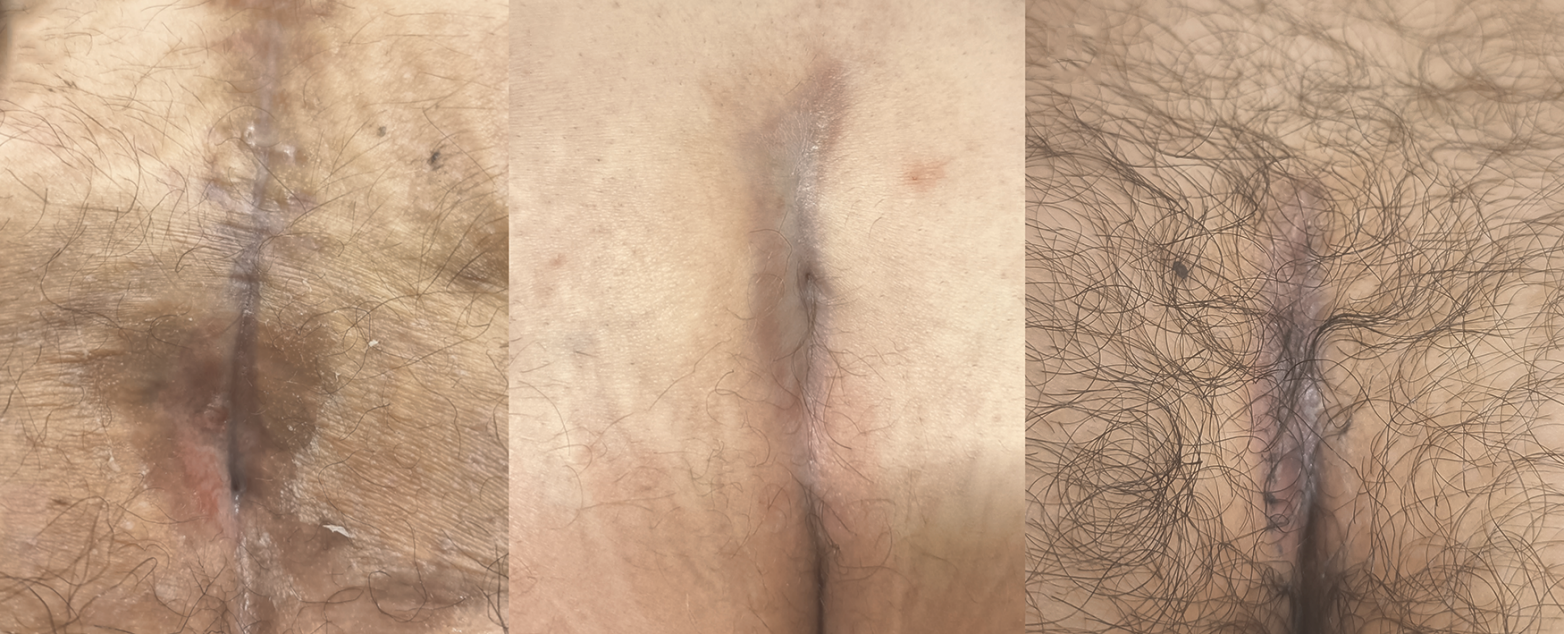
Close-up view of the final results at the 3-month follow-up.

No patients were lost during follow-up at our Outpatient Clinic. No adverse skin reactions or allergies to the product occurred. One case experienced postoperative hyperemia of the skin close to the surgical wound, which was treated with topical ointments. The median time of follow-up was 5 weeks post-surgery (Figure 2). No patients had any physical limitations postoperatively. Six months after the surgery, no recurrence occurred, and all patients reported high satisfaction regarding the cosmetic result (Figure 3).

## Discussion

PD is a common condition affecting adolescents and young adults. Its pathogenesis involves skin injury by the hair follicles, causing a foreign body reaction leading to the formation of an abscess or edema. Its known risk factors include familiarity, repeated local traumatism, sedentary life, and obesity (1, 7). Data on best practices for managing PD without surgery are severely lacking. The only near-universal consensus about PD management is the necessity of incision and drainage of pilonidal abscess as primary treatment. However, recurrence rates after incision and drainage remain high (2, 8). Notably, hygiene and hair removal from the natal cleft and surrounding area plays a crucial role in managing patients with PD and form the cornerstone of almost all care approaches (1, 2, 9).

PD treatment options include phenol or fibrin glue instillation into pits (2, 10), using laser probes to induce tract obliteration (11), subcutaneous tissue radiofrequency ablation (12), classical or minimally invasive sinusectomy (2, 13), endoscopic pilonidal sinus treatment or video-assisted pilonidal sinus ablation (2, 4, 14), and *en bloc* removal of affected tissue followed by primary closure or healing by secondary intention or flap coverage in cases of chronic or multiply recurrent PD (2, 15–18). Removal of affected tissue with *en bloc* resection followed either by primary closure or healing by secondary intention has been the most common definitive surgical PD treatment for decades. However, it is associated with longer healing times and greater pain and disability than less radical procedures.

Several studies reported that minimally invasive techniques might represent the optimal choice for treating PD in the pediatric setting, mainly due to their lower impact on daily activities in the postoperative period compared to open approaches (2, 15). Open surgery remains the treatment preferred in patients with recurrent or chronic PD. However, open healing has a higher recurrence risk (∼26%) and wound healing time (38–92 days) than off-midline primary closure, minimally invasive approaches, or other surgical techniques. No clear advantage has been shown for open healing over primary closure. In addition, when primary closure is the desired surgical option, off-midline closure should be recommended (3, 19). At our institution, we opt for a midline or off-midline wide excision of the pathologic tissue (depending on the singular case) and totally or partially open healing by secondary intention.

ROS play a key role in wound healing. They are secondary messenger-signaling molecules promoting immunocyte recruitment to the wound site and effective tissue repair. They also have a bacteriostatic effect by inhibiting the growth of adjacent contaminating bacteria and play a major role in angiogenesis by stimulating endothelial cell and keratinocyte proliferation and migration. Finally, they accelerate wound healing by promoting collagen synthesis and fibroblast division and migration (20, 21).

**Figure 4:**
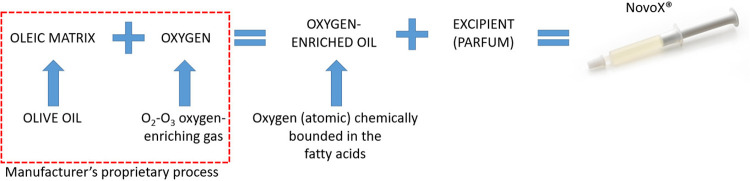
Novox medical device components and manufacturing process.

NovoX is a new oxygen-enriched oil-based product claimed to create a local wound microenvironment unfavorable to pathogen growth. In particular, NovoX's oxygen-enriched oleic matrix (organic extra virgin olive oil) enables the stable and prolonged release of limited ROS amounts and has filmogenic, protective, and barrier actions (Figure 4) (22–24). Notably, it has recently shown encouraging results in treating ulcers, skin lesions, wound dehiscences, and surgical wounds. Interestingly, since the device's effectiveness is prolonged, it only needs to be changed 2–3 times per week (4, 6, 25). Therefore, we tested this new oxygen-enriched oil-based product in a preliminary study on pediatric patients undergoing wound healing by secondary intention after midline open surgery for PD.

## Conclusion

Our experience suggests that this new oxygen-enriched oil-based product (NovoX) is easy to apply, safe, and effective for treating pediatric patients undergoing surgical treatment for chronic PD. In particular, it appears to confer slightly faster wound healing with good aesthetic outcomes in patients treated *via* open surgery followed by healing by secondary intention. Further studies with larger sample sizes are highly encouraged to confirm our preliminary results.

## Data Availability

The original contributions presented in the study are included in the article/Supplementary Material, further inquiries can be directed to the corresponding author/s.
